# Management strategies for the treatment and prevention of postoperative/postdischarge nausea and vomiting: an updated review

**DOI:** 10.12688/f1000research.21832.1

**Published:** 2020-08-13

**Authors:** Ofelia Loani Elvir-Lazo, Paul F. White, Roya Yumul, Hillenn Cruz Eng

**Affiliations:** 1 Department of Anesthesiology, Cedars-Sinai Medical Center, Los Angeles, CA, 90048, USA; 2 The White Mountain Institute, The Sea Ranch, Sonoma, CA, 95497, USA; 3 Instituto Ortopedico Rizzoli, University of Bologna, Bologna, Italy; 4 David Geffen School of Medicine-UCLA, Charles R. Drew University of Medicine and Science, Los Angeles, CA, 90095, USA; 5 Department of Anesthesiology, PennState Hershey Medical Center, Hershey, PA, 17033, USA

**Keywords:** Postoperative nausea and vomiting (PONV), Postdischarge nausea and vomiting (PDNV), Retching, Multimodal antiemetic therapy, Antiemetic drugs, Aromatherapy, Non-pharmacologic antiemetic therapies, Neiguan point (PC6).

## Abstract

Postoperative nausea and vomiting (PONV) and postdischarge nausea and vomiting (PDNV) remain common and distressing complications following surgery. The routine use of opioid analgesics for perioperative pain management is a major contributing factor to both PONV and PDNV after surgery. PONV and PDNV can delay discharge from the hospital or surgicenter, delay the return to normal activities of daily living after discharge home, and increase medical costs. The high incidence of PONV and PDNV has persisted despite the introduction of many new antiemetic drugs (and more aggressive use of antiemetic prophylaxis) over the last two decades as a result of growth in minimally invasive ambulatory surgery and the increased emphasis on earlier mobilization and discharge after both minor and major surgical procedures (e.g. enhanced recovery protocols). Pharmacologic management of PONV should be tailored to the patient’s risk level using the validated PONV and PDNV risk-scoring systems to encourage cost-effective practices and minimize the potential for adverse side effects due to drug interactions in the perioperative period. A combination of prophylactic antiemetic drugs with different mechanisms of action should be administered to patients with moderate to high risk of developing PONV. In addition to utilizing prophylactic antiemetic drugs, the management of perioperative pain using opioid-sparing multimodal analgesic techniques is critically important for achieving an enhanced recovery after surgery. In conclusion, the utilization of strategies to reduce the baseline risk of PONV (e.g. adequate hydration and the use of nonpharmacologic antiemetic and opioid-sparing analgesic techniques) and implementing multimodal antiemetic and analgesic regimens will reduce the likelihood of patients developing PONV and PDNV after surgery.

## Introduction

Nausea is an unpleasant sensation causing discomfort in the stomach area which gives the feeling of the impending need to vomit or retch. It is often a transient sensation which is frequently followed by active retching or tachycardia and increased salivation ^1, 2^. Vomiting is the involuntary, forceful expulsion of the contents of the stomach through the mouth and/or nose ^3, 4^. The incidence of these side effects varies from 30–80% after elective surgery depending on the type of anesthesia and surgery as well as predisposing patient risk factors ^5, 6^.

Postoperative nausea and vomiting (PONV) describes nausea and/or vomiting or retching occurring in the postanesthesia care unit (PACU) or during the first 24–48 hours after surgery ^7^. Postdischarge nausea and vomiting (PDNV) refers to symptoms that occur after discharge from the hospital or surgical care facility ^8^. Not only is PONV a distressing complication from the patient’s perspective but also it can result in dehydration, electrolyte imbalance, acid base imbalance, pulmonary aspiration, pneumothorax, hypoxia, esophageal rupture, increased intracranial pressure, suture rupture, wound dehiscence, bleeding, delay in the ability to resume oral intake, prolonged PACU and/or hospital stay, fatigue, anxiety, unanticipated hospital admission or readmission, and increased medical costs. The distressing symptoms of PONV/PDNV also contribute to patient dissatisfaction with their surgical experience ^9– 12^. PONV prophylaxis is economically beneficial for the hospital when a rational multimodal program is implemented based on patient and procedural risk factors ^13, 14^.

There have been over 4,000 peer-reviewed publications describing treatments for PONV/PDNV in the last 50 years, and numerous new antiemetic drugs and devices have been introduced into clinical practice, yet practitioners have been unable to eliminate this common postoperative problem ^6, 15^. The use of opioid analgesics during the perioperative period for the treatment and/or prevention of pain is a major contributing factor in patients who are at risk of developing PONV and PDNV. Dinges *et al*. ^16^ found that, compared to morphine, the risk ratio for nausea and vomiting did not significantly differ among different opioid compounds except for a higher incidence with buprenorphine and a lower incidence with fentanyl. Despite more widespread use of combinations of prophylactic antiemetic drugs, shorter-acting anesthetic, analgesic, and muscle relaxant drugs, and multimodal analgesic regimens, PONV still affects approximately 30% of all elective surgical patients, with certain high-risk patients experiencing rates of up to 80% ^5, 6, 17^. As newer antiemetic drugs with better safety profiles are introduced into clinical practice, clinical studies are needed to determine the most cost-effective practices for controlling PONV while minimizing other side effects due to unexpected drug–drug interactions. The high incidence of PONV has persisted in part because of the tremendous growth in ambulatory surgery and the increased emphasis on earlier mobilization and discharge after both minor and major operations ^18^. One in four patients undergoing ambulatory laparoscopic surgery experienced PONV before discharge. Also, the combination of PONV and pain was present in more than 50% of this patient population. Of interest, a higher incidence of PONV was reported in patients with longer preoperative waiting times (>45 minutes) ^19^. Despite the extensive literature, the optimal prophylactic antiemetic regimen for specific surgical procedures has not been established ^5, 17, 20^. This review article will focus on the most recently published peer-reviewed literature, as well as some of the classical references, considering both prevention and treatment of PONV using evidence-based multimodal antiemetic prophylaxis regimens. We will also examine pharmacological and nonpharmacological approaches.

## Risk factors for PONV and PDNV

By identifying risk factors for PONV, we can ensure that those patients who are the most in need and stand to gain the greatest benefit receive optimal antiemetic prophylaxis ^21– 26^. A number of factors, including patient-, anesthetic-, and surgical-related factors, influence the occurence of postoperative emetic symptoms ( Table 1– Table 3) ^27, 28^. Patient-specific factors include female gender (odds ratio [OR] 2.57), non-smoking status (OR 1.82), history of PONV or motion sickness (OR 2.09) ^34– 37^, and age <50 years (OR 1.79 in PACU and OR 2.17 for PDNV) ^27, 37, 38^. In children, prior to puberty, female gender does not increase the risk of PONV ^29, 30, 39^. There is very limited literature regarding the risk of POV/PONV in children exposed to secondhand smoke. In children, both a prior history of PONV or postoperative vomiting (POV) and a history of PONV or POV in a parent or sibling increase their risk of POV/PONV ^31^. Chandrakantan *et al*. and Kocaturk *et al*. ^40, 41^ found that when PONV persisted into the postdischarge period, pain was often a contributing factor. Turgut *et al*. ^42^ also reported that PONV was more common in disabled patients younger than 18 years. Younger children were at lower risk of PONV ^29, 43– 45^, while others have found no effect of age on PONV in children ^46^.

**Table 1.** Postoperative nausea and vomiting (PONV) risk factors in adults related to patient, anesthesia, and surgery.

Category	Risk factors in adults
**Patient related**	Female gender History of PONV Motion sickness Non-smoking status Age <50 years
**Anesthesia related**	Anesthesia technique (general anesthesia results in higher incidence of PONV than does regional anesthesia) Prolonged duration of anesthesia Volatile agents Nitrous oxide (>50%) Intraoperative and postoperative opioid analgesics Increased doses of neostigmine (>3 mg)
**Surgery related**	Extended surgical procedures Surgery categories (e.g. neurosurgery, laparoscopic surgery, cholecystectomy, intra-abdominal surgery, and gynecological surgery)

**Table 2.** Patient-, anesthesia-, and surgery-related risk factors for postoperative nausea and vomiting (PONV) in children.

Category	Risk in children (Eberhart classification) ^29^
**Patient related**	History of postoperative vomiting (POV) or PONV in relatives Age >3 years: it is rare in children <3 years old, increases with age over 3, and decreases again with puberty ^29^
**Surgery related**	Type of surgery: strabismus ^30^ Duration of surgery >30 minutes

The risk of POV for children with 0 to 1, 2, 3, or 4 of these risk factors is associated with an incidence of PONV of 10, 30, 50, and 70%, respectively. This scoring system has also been validated for children having surgery other than strabismus surgery; POV was observed in 3, 11, 30, and 40% of children who had 0, 1, 2 or 3 risk factors, respectively ^31^.

**Table 3.** Simplified risk-score ^8^.

**A**	**PONV in adults** ^8^	**Points**	
	Female gender	1	(A) When 0, 1, 2, 3, or 4 of the depicted independent predictors are present, the corresponding risk of PONV is approximately 10, 20, 40, 60, or 80%, respectively
	Non-smoker	1	
	History of PONV	1	
	Postoperative opioids	1	
	**Maximum score**	**4**	
**B**	**POV in children**		
	Surgery >30 minutes	1	(B) When 0, 1, 2, 3, or 4 of the depicted independent predictors are present, the corresponding risk of PONV is approximately 10, 10, 30, 50, or 70%, respectively
	Age >3 years	1	
	Strabismus surgery	1	
	History of POV or PONV in relatives	1	
	**Maximum score**	**4**	
**C**	**PDNV in adults**		
	Female gender	1	(C) When 0, 1, 2, 3, 4, and 5 risk factors are present, the corresponding risk for PDNV is approximately 10, 20, 30, 50, 60, and 80%, respectively
	History of PONV	1	
	Age <50 years	1	
	Use of opioids in the PACU	1	
	Nausea in the PACU	1	
	**Maximum score**	**5**	
**D**	**PDV in children** ^32, 33^		
	Strabismus, tonsillectomy, and dental surgery	1	(D) PDNV incidence of 11–14% in outpatient pediatric patients Long-acting opioids in operating room as well as during postdischarge had the highest incidence of PDNV at 36%
	Intraoperative or postdischarge opioids	1	
	Long-acting intraoperative opioids	1	
	Pain	1	
	Presence of nausea on discharge	1	
	**Maximum score**	**5**	

PACU, postanesthesia care unit; PDNV, postdischarge nausea and vomiting; PDV, postdischarge vomiting; PONV, postoperative nausea and vomiting; POV, postoperative vomiting

Anesthesia-related risk factors ( Table 1– Table 3) include the use of opioids, volatile agents, nitrous oxide (which increases the risk for postoperative vomiting), and high doses of neostigmine for the reversal of residual neuromuscular blockade ^32, 38, 47– 51^. General anesthesia is associated with a higher incidence of PONV compared with regional anesthesia ^35, 52, 53^ secondary to the greater requirement for opioid medication to control postoperative pain after general anesthesia in both adults and children ^35, 54^. The performance of peripheral nerve blocks ^55– 58^, ganglion block ^59^, and wound infiltration with local anesthetic ^60^ has been shown to decrease the incidence of PONV. Surgery-related predictors include prolonged surgical procedures, with each 30 minutes increasing the risk of PONV by 60% ^36^. Certain types of surgery (e.g. ophthalmic, oral, and maxillofacial surgeries, ENT surgery, neurosurgery, laparoscopy, abdominal surgeries, cholecystectomy, and gynecological surgery) have a higher incidence of PONV perhaps because of the longer exposure to general anesthesia and use of larger doses of opioid medications. In open abdominal or intra-abdominal laparoscopic surgery, post-operative ileus can occur because of gut ischemia releasing 5-HT ^8, 38^. Opioid use is related to a number of perioperative side effects, one of which is PONV, and they can hinder hospital discharge and return to normal activities of daily life after surgery ^61, 62^. Li *et al*. demonstrated that non-smoking female patients who exhibited a fentanyl-induced cough at anesthesia induction also had a higher likelihood of developing PONV ^63^. In a retrospective observational study, Hozumi and colleagues found a dose-dependent relationship between the dosage of remifentanil administered during surgery and an increased risk of developing PONV ^64^. Strategies to minimize the use of opioids should be considered for all patients at moderate and high risk of developing PONV. Although the notion is still controversial, some studies have suggested that the risk of PONV is higher with some opioids (e.g. morphine) than others (e.g. hydromorphone) ^65^. Palumbo *et al*. ^66^ found that compared to remifentanil, fentanyl was associated with a higher incidence of PONV after inguinal hernia repair. Tao *et al*. ^67^ reported that the incidence of PONV in gynecological patients who underwent laparoscopic surgery was lower when using intraoperative and postoperative intravenous (IV) oxycodone compared to IV sufentanil. However, Han *et al*. ^68^ did not find a difference in the incidence of PONV when IV oxycodone was compared to IV sufentanil in the PACU, but on the post-surgical wards a higher incidence of PONV was found in patients receiving sufentanil ^69^. The use of long-acting opioid analgesic techniques like intrathecal morphine or modified-release oral opioids not only prolongs the duration of analgesia but also can extend the duration of PONV ^70– 72^. In one study, naloxone was added to intrathecal morphine and significantly decreased the severity of postoperative nausea and pruritus after cesarean section ^73^.

The use of propofol for anesthesia (or sedation) is associated with a 3.5-fold reduction in the incidence of PONV in adults and 5.7-fold reduction in children ^74^. Bhakta *et al*. ^75^ suggested that propofol-based anesthesia (e.g. total IV anesthesia [TIVA]) was associated with significantly less POV and faster recovery compared to standard “balanced” anesthesia in patients undergoing gynecological laparoscopy. Etomidate has been shown to produce an increase of PONV compared to propofol in elderly patients undergoing gastroscopy and ambulatory surgery ^76– 80^. Ketamine has morphine-sparing effects in lower subanesthetic dosages ^81^; however, its psychosomatic effects with high dosages during dissociative anesthesia (and sedation) have led to emergence agitation and PONV ^82^. Pan *et al*. ^83^ found that ketamine (0.5–1.0 mg/kg intra-articular or 0.01–0.15 mg/kg IV) did not increase PONV in patients undergoing knee arthroscopy. Perioperative intravenous ketamine minimally reduced the risk of PONV (high‐quality evidence) ^84^. Moro *et al*. ^85^ compared saline to ketamine 0.2–0.4 mg/kg in patients who underwent laparoscopic cholecystectomy and found that the incidence of PONV did not differ. Controversial findings have suggested that ketamine and etomidate did not increase PONV at doses commonly administered for induction of anesthesia ^86^ and that low-dose ketamine may actually reduce PONV by decreasing postoperative opioid requirements ^87, 88^.

A history of chemotherapy-induced nausea and vomiting (CINV) may increase the risk of PONV after surgery (OR 3.15) ^89^. Psychological factors such as acute anxiety sensitivity (i.e. a fear of behaviors or sensations associated with the experience of anxiety) ^90^ should be added to PONV risk-scores, and prophylaxis should be considered when patients show evidence of high anxiety sensitivity. Odom *et al*. ^91^ found that the psychometric properties of the Ambulatory Surgery Index of Nausea, Vomiting, and Retching (AS-INVR) provided a reliable and valid measure of the amount of distress and nausea and vomiting. Ethnicity and genetic polymorphisms ^92– 98^ could be useful in improving the predictability of PONV, which would help to improve both the prevention and the treatment of PONV. For example, CYP2D6 seems to be related to a higher incidence of PONV, especially in the first 24 hours after surgery. The ABCB1 transporter could reduce PONV owing to its association with the effectiveness of ondansetron in antiemetic prophylaxis. With regard to ethnicity, the incidence of PONV is known to be higher in non-Africans than in Africans undergoing the same surgical procedures with the same anesthetic drugs ^92– 98^. Interestingly, the platelet count (PLT), mean platelet volume (MPV), and MPV/PLT ratio were used to predict POV in children ^99^. The neutrophil/lymphocyte ratio (NLR) was also used to predict PONV: when the NLR was greater than 2 in patients undergoing ambulatory maxillofacial surgery, they experienced a statistically higher incidence of PONV compared to an NLR of less than 2 ^100^.

## Risk-scoring systems for PONV and PDNV

Antiemetics produce major side effects ranging from mild headache to severe arrhythmia due to QTc prolongation. Therefore, it is essential to calculate the risk of developing PONV and PDNV in each patient to reduce excessive use of antiemetics for prophylaxis ^101, 102^. Apfel *et al*. ^103 ^developed a simplified risk-scoring system for PONV in adults; the primary predictors consist of female gender, history of PONV or motion sickness, non-smoking status, and postoperative opioid use ( Table 1). The PONV risk increases by 10, 21, 39, 69, and 79% when 0, 1, 2, 3, and 4 factors are present, respectively. The use of Apfel's risk-scoring system is more sensitive and specific compared to predicting PONV based on history of PONV or type of surgery alone ^103, 104^.

However, the adult risk-scores are not directly applicable to children ^30^. The Eberhart classification scoring system is commonly used for children and includes the following risk factors ^29^: age >3 years, duration of surgery >30 minutes, strabismus surgery, and history of POV or a close relative with POV/PONV. The risk of POV for children undergoing strabismus surgery with 0 to 1, 2, 3, or 4 of these risk factors was 10, 30, 50, and 70%, respectively. This scoring system has also been validated for children having surgery other than for strabismus, and POV was observed in 3, 11, 30, and 40% of children who had 0, 1, 2, or 3 risk factors, respectively ^31^. A study by White *et al*. ^27^ reported that an Apfel risk-score of 3 or 4 (versus a score of 1–2) is associated with a higher incidence of emesis in the first 24 hours after surgery irrespective of administration of multiple antiemetics as prophylaxis.

The prevention of PONV should be tailored to the patient’s risk-score to avoid side effects and unnecessary costs related to administering multiple antiemetic drugs irrespective of their risk ^105– 107^. The prevention of PDNV is still a problem in the ever-increasing group of outpatients having more complicated ambulatory and office-based surgical procedures ^108, 109^. In a multi-center study, 37% of 2,170 adult ambulatory surgery patients administered general anesthesia exhibited PDNV ^110^. Since these patients often do not have ready access to “rescue” antiemetic drug therapies after their discharge home, the use of simple nonpharmacologic antiemetic devices (e.g. acupressure) represents a low-risk and cost-effective alternative ^108, 109^. White *et al*. ^111^ used the Pressure Right acupressure device in combination with antiemetic drugs to significantly reduce the incidence of vomiting from 0–72 hours after surgery with an associated improvement in patient satisfaction with their PONV management. Coloma *et al*. ^112^ reported that the use of acustimulation with the ReliefBand can be used as an alternative to ondansetron for the treatment of established PONV. However, the use of ondansetron (4 mg IV) in combination with the ReliefBand device improved the overall response rate compared to acustimulation alone. Similar results were reported by White and colleagues ^113^. Odom-Forren *et al*. found that pain and postdischarge opioid use seem to be factors in late PDNV ^114^. The main difference between risk factors for PONV and PDNV was that patients who experienced nausea in the PACU had a threefold greater risk for developing PDNV ^115^. Interestingly, non-smoking status was not an independent predictor for PDNV. When 0, 1, 2, 3, 4, and 5 risk factors are present, the corresponding risk for PDNV is approximately 10, 20, 30, 50, 60, and 80%, respectively ^110^.

## Perioperative antiemetic drugs used for the treatment and/or prevention of PONV

The concern with widespread prescribing of anti-vomiting drugs is primarily related to the increased costs associated with this practice, especially when expensive proprietary antiemetics are prescribed. In addition, side effects and adverse drug interactions associated with the routine use of prophylactic antiemetics is another concern (e.g. extrapyramidal effects, sedation, arrhythmias, orthostatic hypotension) ^116– 118^. The side effects related to the routine use of prophylactic antiemetic drugs (e.g. restlessness, dry mouth, drowsiness, headache, tachycardia, hypotension, and fatigue) can also prolong the length of stay in the surgical facility and the time to restart routine activities of daily living ^119– 121^.

### Antiemetic drug classes

The currently available antiemetic drugs for the treatment and prevention of PONV include the 5-hydroxytryptamine (5-HT _3_) receptor antagonists, neurokinin-1 (NK-1) receptor antagonists, corticosteroids, butyrophenones, metoclopramide, phenothiazine, prochlorperazine, antihistamines, and anticholinergics ( Table 4). Conventional prophylactic dosages and suggested timings for the administration of antiemetics are listed in Table 5. Apfel *et al*. reported that droperidol, dexamethasone, and ondansetron all carry similar antiemetic efficacy when given for prophylaxis ^122^.

**Table 4.** Receptor site affinity of available antiemetic drugs.

Drug group	Dopamine (D2)	Muscarinic cholinergic	Histamine (H2)	Serotonin (5-HT3)	NK-1 antagonist	CB-1 modulator	MOR antagonist	Gaba mimetic
**Antiserotonin**								
Ondansetron	-	-	-	++++	-	-	-	-
Granisetron	-	-	-	++++	-	-	-	-
Tropisetron	-	-	-	++++	-	-	-	-
Palonosetron	-	-	-	++++	-	-	-	-
**Phenothiazines**								
Fluphenazine	++++	+	++	-	-	-	-	-
Chlorpromazine	++++	++	++++	+	-	-	-	-
**Butyrophenones**								
Droperidol	++++	-	+	+	-	-	-	-
Haloperidol	++++	-	+	-	-	-	-	-
Domperidone	++++	-	-	-	-	-	-	-
**Antihistamines**								
Diphenhydramine	+	++	++++	-	-	-	-	-
Promethazine	++	++	++++	-	-	-	-	-
**Anticholinergics**								
Scopolamine	+	++++	+	-	-	-	-	-
**Benzamides**								
Metoclopramide	+++	-	+	++	-	-	-	-
**Tricyclic antidepressants**								
Amitriptyline	+++	+++	++++	-	-	-	-	-
Nortriptyline	+++	++	+++	-	-	-	-	-
**Neurokinin-1**								
Aprepitant	-	-	-	-	++++	-	-	-
Fosaprepitant	-	-	-	-	++++	-	-	-
**Others**								
Dronabinol	-	-	-	-	-	++++	-	-
Nabilone	-	-	-	-	-	+++	-	-
Naloxone	-	-	-	-	-	-	++++	
Lorazepam	-	-	-	-	-	-	-	+++

The number of positive signs (+) indicates receptor activity. This table was adapted with permission from White PF (ed). Ambulatory Anesthesia and Surgery. London, WB Saunders, 1997 Page 442 ^133^

**Table 5.** Prophylactic doses and timing for the administration of antiemetic drugs.

Drug group	Drugs	Dose	Timing	Adverse effect
**Serotonin (5-HT _3_** **receptor) antagonists**	Ondansetron	4–8 mg intravenously (IV) every 4–8 hours	End of surgery	Headaches, constipation, flushing, fatigue, malaise, raised liver enzymes
	Granisetron	1–2 mg IV		
	Ramosetron	0.3 mg IV 0.1 mg PO		
	Palonosetron	0.075–0.25 mg IV		
**Corticosteroids**	Dexamethasone	4–10 mg IV	After induction of anesthesia	Elevated blood glucose level, diabetes mellitus, hypotension/hypertension
**Butyrophenone**	Droperidol	0.625–1.25 mg IV	After induction of anesthesia	Psychomimetic effects, extrapyramidal side effects, Parkinson’s disease, sedation, lightheadedness, prolonged QT interval
**Neurokinin** **antagonists (NK-1** **receptors)**	Aprepitant	40 mg orally	1–2 hours prior to induction	Headaches, constipation, fatigue
	Fosaprepitant	150 mg IV	After induction of anesthesia	
**Anticholinergics**	Scopolamine	Transdermal patch 0.3–0.6 every 24 hours	Evening prior to surgery or in preoperative period	Dizziness, dry mouth, visual disturbances, tachycardia, confusion, urinary retention
**Dopamine antagonists**	Metoclopramide	10–25 mg IV	15–30 minutes prior to end of surgery	Sedation, hypotension (fast injection), headache, extrapyramidal symptoms
	Amisulpride IV	5–10 mg IV	At induction of anesthesia	

***5-HT _3_ receptor antagonists***. 5-HT _3_ receptor antagonists are recommended as the first-line regimen for PONV prophylaxis. Ondansetron IV is commonly administered near the end of surgery. Multiple trials have reported that ondansetron 4 mg IV (usually administered before the end of surgery) was effective to prevent and treat PONV, facilitating both early and late recovery and improving patient satisfaction after different types of surgery (e.g. outpatient laparoscopy ^123^, laparoscopic surgery ^124– 126^, major surgical procedures in women ^127^, and cesarean section ^128^). When ondansetron was administered at 8 mg, a reduction in postpartum headache up to 4 days was observed; it also reduced PONV as it did with 4 mg ^128^. Koyuncu *et al*. ^129^ found that ondansetron 8 mg decreased the analgesic effect of acetaminophen 1 g (then 1 g every 6 hours for 24 hours) during the initial postoperative period after hysterectomy. Granisetron, a more selective 5-HT _3_ antagonist, has been suggested to provide more sustained antiemesis as a prophylactic ^124^. White *et al*. showed that granisetron (1 mg orally) was just as effective as ondansetron (4 mg IV) for lowering the occurrence of PONV after laparoscopic procedures ^124^. Granisetron has been reported to be effective alone or in combination to treat PONV in patients undergoing laparoscopic surgery ^130– 132^. Ramosetron has higher affinity to the 5-HT _3_ receptor and longer duration of antiemetic action, resulting in a similar or greater prophylactic antiemetic effect than the older 5-HT _3_ receptor antagonists (e.g. granisetron and ondansetron) and has also been reported to provide a better prophylactic and antiemetic efficacy in patients at moderate to high risk ^96, 134– 143^.

Palonosetron is a second-generation 5-HT _3_ receptor antagonist with proposed higher efficacy and more prolonged duration of action when used for antiemetic prophylaxis ^144^. Palonosetron was found to be more efficient than ondansetron or ramosetron for antiemetic prophylaxis in patients undergoing laparoscopic surgery ^145– 147^. However, Kim *et al*. ^148^ failed to find a difference between palonosetron and ramosetron in patients undergoing any type of elective surgery involving general or regional anesthesia.

***Glucocorticoid steroids***. Dexamethasone, a corticosteroid, has been shown to be an effective antiemetic when administered at a dosage of 4–12 mg IV ^149– 151^. However, Ormel *et al*. ^152^ found that dexamethasone 4–5 mg was equally as effective as 8–10 mg in terms of antiemetic efficacy. Dexamethasone antiemetic efficacy alone or in combination has been reported in patients undergoing laparoscopic cholecystectomy ^153– 164^, other abdominal laparoscopic procedures ^150^, breast cancer surgery ^165^, large and small bowel surgery ^166^, total knee arthroplasty ^167^, joint replacement surgery ^168^, gynecological laparoscopic procedures ^169, 170^, cesarean delivery ^171^, scoliosis correction surgery ^172^, vitrectomy under local anesthesia ^173^, and upper extremity surgery ^174^ as well as in endoscopic adenoidectomy ^175^ and strabismus correction surgery ^176^ in children. Interestingly, there are reports that dexamethasone did not reduce PONV in patients undergoing surgery for facial fracture ^177^, laparoscopic surgery for suspected appendicitis ^178^, and microvascular decompression surgery of the trigeminal nerve root ^179^. Singh *et al*. ^180^ concluded that dexamethasone has equal antiemetic efficacy compared to 5-HT _3_ receptor antagonists up to 24 hours after surgery. Concerns remain regarding potential complications (e.g. delayed wound healing, hyperglycemia, and risk of infections) in “at-risk” patient populations (e.g. diabetics) ^122, 181^.

Betamethasone has also been shown to be an effective antiemetic. Aasboe *et al*. ^182^ compared betamethasone 12 mg intramuscularly (IM) to saline when administered 30 minutes before the start of surgery and reported that betamethasone reduced both postoperative pain and nausea in outpatients undergoing ambulatory foot (hallux valgus) surgery or hemorrhoid procedures. Comparable results were attained in patients undergoing ambulatory surgery ^183^ and elective breast surgery ^184^ and in high-risk cardiac surgical patients ^185^. However, in a placebo-controlled study by Nordin *et al*. ^186^ comparing betamethasone 8 mg *per os* (PO) and betamethasone 8 mg IV when administered 1 hour before induction of anesthesia in patients undergoing elective Roux-y-gastric bypass, betamethasone was of limited benefit in preventing PONV.

***NK-1 receptor antagonists***. NK-1 receptor antagonists with long elimination half-life values are effective for the prophylaxis and treatment of PONV ^187^. Gesztesi *et al*. ^188^ found that the NK-1 receptor antagonist CP-122,721 (200 mg PO) decreased emetic episodes compared with ondansetron (4 mg IV) during the first 24 hours after gynecologic surgery. The NK-1 receptor antagonist aprepitant appears to be more effective in decreasing PONV as compared with ondansetron ^189, 190^. Aprepitant alone or in combination was associated with a low overall incidence of PONV ^191, 192^ in patients undergoing laparoscopic surgery ^193^, craniotomy ^194^, mastectomy and thyroidectomy ^195^, and elective surgery ^196^ and in pediatric patients ^197^. Because of its high cost, aprepitant should be used only in patients at high risk of developing PONV and in those who could experience serious adverse outcomes due to PONV as well as in patients who may have side effects from less-expensive antiemetic drugs ^191, 192^.

Fosaprepitant 150 mg IV, a water-soluble lipid formulation of the NK-1 receptor antagonist, was compared to IV ondansetron 4 mg when administered before induction of anesthesia in patients with a moderate-to-high risk of PONV (Apfel simplified score ≥2) undergoing general anesthesia ^197^, obtaining a greater decrease in the incidence of vomiting during the first 48 hours after surgery. Similar results were reported in patients undergoing craniotomy ^198^ and gynecologic abdominal surgery with patient-controlled epidural analgesia ^199^.

***Butyrophenone***. Droperidol, which acts on central dopamine receptors, is a highly cost-effective antiemetic treatment, regardless of the risk of extrapyramidal side effects and the potential for prolonging the electrocardiographic QT interval ^200^. Multiple well-controlled, randomized, comparative clinical trials have validated droperidol to be as safe and effective as the more costly 5-HT _3_ and NK-1 antagonists ^201, 202^. There is minimal to no clinical significance in the degree of QT-interval prolongation correlating to antiemetic doses of the drug ^203^. The risk of QTc prolongation was actually decreased by administrating a combination of droperidol and a 5-HT _3_ receptor antagonist ^204^. The combination of dexamethasone, ondansetron, and droperidol is highly efficacious in preventing PONV in adults. Clinical studies have stated the efficacy of droperidol in reducing PONV in different surgical procedures such as tonsillectomy in children ^205^ and ambulatory surgery ^206^. Nevertheless, Bourdaud *et al*. ^207^ compared the efficacy of a combination of ondansetron (100 µg/kg, IV), dexamethasone (125 µg/kg, IV), and droperidol (50 µg/kg, IV) in pediatric patients at high risk of POV and concluded that adding droperidol to a prophylactic combination of ondansetron and dexamethasone did not decrease the incidence of POV below that obtained with the two drugs alone, though the addition of droperidol increased the risk of drowsiness. Singh *et al*. ^208^ reported that haloperidol was equivalent to the popular 5-HT _3_ receptor antagonists in preventing vomiting on the first day after surgery. The incidence of QTc prolongation with haloperidol is statistically equivalent to the 5-HT _3_ antagonists. Brettner *et al*. ^209^ found gender-specific differences in the incidence of PONV (female > males) in the PACU after low-dose haloperidol (0.5 mg IV).

***Dopamine antagonists and gastrokinetic drugs***. Metoclopramide is one of the most utilized antiemetics for treating PONV when 5-HT _3_ antagonists, dexamethasone, and/or droperidol prophylaxis is unsuccessful. A systematic review reported that in patients undergoing cesarean delivery under neuraxial anesthesia, the use of metoclopramide 10 mg IV was effective and safe for the prevention of early PONV ^210^. Amisulpride has been found to be effective in treating PONV after failed prophylaxis ^211^ in treating patients at low-to-moderate risk of PONV who received no prior PONV prophylaxis ^212^, patients at moderate-to-high risk ^213^, or patients at high risk of PONV who developed emetic symptoms after prophylaxis with ondansetron or dexamethasone ^214^.

***Anticholinergics***. Scopolamine is a centrally active anticholinergic drug and can be as efficacious as droperidol (1.25 mg) or ondansetron (4 mg) in reducing PONV in the early and late postoperative periods. Nonetheless, there are concerns about using it routinely for antiemetic prophylaxis because of its slow onset of action and adverse effects (e.g. dry mouth, drowsiness, and visual disturbances) ^215^. Despite this, scopolamine remains a suitable and cost-effective substitute to ondansetron in multimodal treatment prophylaxis in patients with motion-induced emesis and high-risk patients undergoing major operation ^215^. Apfel *et al*. ^216^ reported that transdermal scopolamine (TDS) was associated with significant reductions in PONV during the first 24 hours after anesthesia. TDS was also associated with a higher prevalence of visual disturbances at 24–48 hours after surgery. Pergolizzi *et al*. ^217^ concluded that TDS significantly reduces PONV/PDNV in many different types of surgical patients, and it is recommended in guidelines as a first-line or second-line prophylactic antiemetic. Kassel *et al*. ^218^ concluded that scopolamine should be reconsidered as a routine agent for PONV prevention in the general surgical population but should be avoided in both pediatric and elderly surgical populations.

***Drugs with opioid-sparing effects contributing to antiemetic activity***. Neuromodulator drugs such as tricyclic antidepressants, gabapentin, olanzapine, mirtazapine, benzodiazepines, clonidine, and cannabinoids have been reported to be effective in preventing nausea and vomiting as a result of their opioid-sparing effects ^219^. Dexmedetomidine has demonstrated opioid-sparing effects in elderly patients undergoing epiduroscopy ^220^, in patients with a high risk of PONV following gynecological laparoscopic surgery ^221^, and in patients undergoing other types of surgical procedures ^222^. However, dexmedetomidine has produced side effects (e.g. bradycardia and hypotension). Gabapentin has been reported to produce anti-nauseant effects in various clinical settings owing to opioid-sparing effects ^223– 228^. However, White *et al*. found that preoperative pregabalin failed to decrease either PONV or postoperative pain after major gynecologic surgery ^229^. The administration of dimenhydrinate is limited because of its adverse events profile (e.g. dizziness, sedation, and dry mouth, throat, and nose). Kizilcik *et al*. ^230^ found that the dexamethasone-dimenhydrinate combination was effective for PONV prophylaxis. Mirtazapine, a 5-HT _3_ receptor antagonist capable of blocking adrenergic receptors, has been shown to be effective for PONV prophylaxis and to decrease nausea and vomiting in patients after a variety of surgical procedures ^231, 232^. Midazolam, a short-acting benzodiazepine, has been reported to reduce the incidence of PONV and provide an anxiolytic effect in patients undergoing cholecystectomy, appendectomy, gynecological surgery, middle ear surgery, thyroidectomy, and intragastric balloon placement ^233– 241^. Antipsychotic therapies including olanzapine, aripiprazole, and risperidone have been reported to reduce the need for antiemetic medication in the PACU ^242^. Kang *et al*. ^243^ reported that the combination of palonosetron 0.075 mg and the muscle relaxant reversal drug sugammadex 2 mg/kg reduced the incidence of PONV in patients undergoing microvascular decompression. Acetaminophen preoperatively has been associated with a reduced incidence of PONV in children undergoing strabismus surgery ^244^. However, Roberts *et al*. ^245^ found that children who received IV acetaminophen were more likely to experience PONV. Nabilone, a synthetic cannabinoid, has proven clinical utility in chemotherapy-related nausea and vomiting and PONV. However, oral nabilone 0.5 mg (versus placebo) in patients with preoperative risk of PONV greater than 60% ^246^ was reported to be ineffective when given prior to surgery. The addition of nalbuphine (0.5 mg) reduced the incidence and severity of PONV and pruritus after cesarean delivery ^247^. Perioperative intravenous lidocaine infusion (1–5 mg/kg/hour or 2–4 mg/kg/hour) has been reported to reduce PONV, pain scores, perioperative opioid consumption, and duration of hospital stay and accelerate the restoration of bowel function ^248– 257^. However, Dewinter *et al*. ^258^ failed to confirm these findings, concluding that systemic lidocaine had no analgesic effect when added to an opioid-based anesthetic regimen for arthrodesis procedures. Clonidine is an alpha 2 adrenergic agonist which has been used both orally and via neuraxial administration as an adjuvant for the treatment of pain and PONV in a wide variety of surgical procedures (e.g. breast, thyroid, and lower abdominal surgery and laparoscopic cholecystectomy in adults and strabismus surgery in children). Clonidine has been shown to improve pain, reduce morphine consumption, decrease PONV, reduce postoperative shivering, and improve hemodynamic and sympathetic stability ^259– 264^. However, there are also published studies which have contradicted these findings ^72, 265– 268^. Some also reported higher Ramsay sedation scores with clonidine ^266^.

Adequate IV fluid hydration is an effective strategy for decreasing the baseline risk for PONV. It has been suggested that early rehydration in surgical patients with prolonged fasting decreases PONV ^269^. Studies have reported that the administration of perioperative IV colloid ^270^, perioperative IV crystalloid ^271, 272^, and Ringer's lactate (30 mL/kg/hour) ^273^ and early postoperative oral fluid intake ^274^ were associated with a lower incidence of PONV. It has also been suggested that the administration of a perioperative infusion of dextrose reduces PONV ^275^. Nevertheless, two meta-analyses ^276, 277^ reported that perioperative IV dextrose did not reduce the risk for PONV but was effective in reducing the need for antiemetic rescue medications after general anesthesia.

Ginger root is an herbal compound which contains gingerol (Ginjervel) and shogaol (Chagall), which reduce stomach contractions and increase the activity of the gastrointestinal tract and motility due to anticholinergic and antiserotonergic actions, increasing gastric emptying ^278– 280^. Ginger possesses antiserotonergic activity and has free radical scavenging effects on free radicals that induce vomiting ^281, 282^. Ginger is safe and well tolerated, which appears to be useful in both pregnancy ^283– 285^ and chemotherapy-induced PONV ^286^, reducing the need for antiemetic rescue medications ^287– 297^. Ginger has also reduced PONV in patients undergoing cholecystectomy ^287– 289^, nephrectomy ^282, 291^, gynecologic/obstetric surgery ^293– 295^, ambulatory surgery ^292^, cataract surgery ^298^, and thyroidectomy ^299^. However, there are also several studies that have reported contradictory results ^300– 302^. Alcohol pads containing isopropyl alcohol, when applied under the nose, are a highly cost-effective treatment for transient PONV in adults and children ^303– 307^.

Aromatherapy such as essential oils (i.e. spearmint, peppermint, ginger, lavender, and blended orange and peppermint) has also been demonstrated to provide benefits in reducing PONV and PDNV when added to a standard antiemetic treatment regimen ^296, 308– 313^. However, there are other authors who have not found any evidence that aromatherapy decreases PONV ^314– 318^.

## Nonpharmacologic therapies for PONV and PDNV

A wide variety of nonpharmacologic techniques have been used to control emetic symptoms in the postoperative period alone or in combination, including acupressure ^319, 320^, acupuncture ^321, 322^, and transcutaneous electrical nerve stimulation (TENS) ^113, 323– 325^. TENS combined with a wristband pressing on Neiguan P-6 acupoint was effective in preventing PONV after laparoscopic cholecystectomy ^326^. White *et al*. reported that TENS and ondansetron was effective in PONV prophylaxis ^113^. These results were later confirmed when acustimulation was shown to possess analgesic effects ^327^. The adjunct use of the Pressure Right acupressure device was shown to improve the emetic potency of commonly used drugs for antiemetic prophylaxis (e.g. ondansetron, droperidol, and dexamethasone) after major laparoscopic surgery ^115^. Lee *et al*. described the use of P-6 acupoint stimulation for PONV as superior to non-acupoint or sham treatments in reducing PONV and need for rescue antiemetic treatment in the postoperative period ^328^. Acupuncture at ear acupoint alone or in combination with stimulation at the wrist (P-6 acupoint) has been found to be an effective treatment to reduce PONV ^329– 331^. Similar results with dry cupping at the P-6 acupoint ^332^ and preoperative electro-acupuncture ^333^ were found. The Society for Ambulatory Anesthesia guidelines ^38^ mentioned that stimulation of the P-6 acupoint is an effective complementary method to reduce PONV. Other studies support the beneficial effect of P-6 acupoint stimulation in reducing PONV and the need for rescue antiemetics ^112, 334– 337^. Acupoints such as Laogong (PC8), Waiguan (SJ5), Zusanli (ST36), Hegu (LI4), and Quchi (LI11) can be used for reducing PONV as well ^338– 349^. In the pediatric population, acupuncture, electroacupuncture, and laser acupuncture at the P-6 acupoint have all been used to prevent POV after tonsillectomy and/or adenoidectomy, hernia repair, circumcision, orchidopexy, chemotherapy, and strabismus procedures ^321, 350– 362^. However, there are other studies that used these modalities in both adults and children with negative results ^363– 366^. Chewing gum was also associated with a lower incidence of postoperative ileus and PONV ^367– 370^; however, Ge *et al*. ^371^ found no difference.

Music therapy has been alleged to decrease patient anxiety, pain, and emesis, hospital length of stay, and fatigue after surgeries such as hernia repair, coronary angiography, valve replacement, cardiac surgery, breast surgery, elective cesarean section, sigmoidoscopy, colonoscopy, knee arthroplasty, hand surgery, cystoscopy, hysterectomy, gynecological surgery, varicose vein surgery, general abdominal surgery, laparoscopic cholecystectomy, and urological procedures ^336, 372– 389^. However, other studies showed that music therapy did not significantly reduce PONV ^390, 391^. Other alternative modalities such as foot massage were reported to decrease pain and incidence of nausea and improve blood circulation in patients who underwent laparoscopic cholecystectomy ^392^. Frozen ice pops reduced PONV in patients at high risk of PONV who were undergoing major joint replacement surgery ^393^. Another important therapeutic goal for PONV prophylaxis is to avoid surgical oxygen desaturation and maintain muscular tissue oxygen saturation at >70% of the baseline values ^394^ and a normal cerebral oxygen saturation ^395^. These nonpharmacologic alternative therapies can produce additive effects to standard antiemetic drugs without increasing side effects or producing adverse drug interactions.

## Preventing PONV and PDNV through multimodal prophylaxis

PONV has multiple factors contributing to its etiology, leading to an increased awareness in the use of combined therapies that incorporate more than two strategies depending on the overall risk for any given patient ^396^. Multimodal interventions not only reduce PONV but also, more importantly, enhance patient comfort after surgery ^58^. There is no evidence to date to suggest that any one specific antiemetic therapy is especially effective for a particular patient profile or operation. Therefore, a combination of antiemetic drug therapies that act at different neuroreceptor sites has been recommended for at-risk patients ^397^. Some clinicians used a simple method involving the administration of one antiemetic medication for each of the Apfel PONV risk factors. It is commonly accepted that increasing the number of administered antiemetics from one to three improves PONV prophylactic benefit for higher risk patients ^122^. Clinical research has shown combining prophylactic antiemetic drugs can lower the rate of PONV and PDNV occurrence as well as improve the patient’s satisfaction with and assist in their recovery in comparison to using a single antiemetic drug ^9, 398^. Implementation of PONV guidelines and the assessment of the risk of PONV using the Apfel scoring system reduced the incidence of PONV in patients undergoing ambulatory breast surgery and improved anesthesia providers' compliance with a preoperative PONV risk assessment ^61, 399– 402^. A combination therapy with antiemetic drugs acting at separate receptor sites should be provided to patients with moderate-to-high risk for PONV ^396^. A multimodal approach provides a considerable decrease in the incidence of PONV to less than 10% along with an increase in patient satisfaction and reduced side effects ^203, 403^.

Bruderer *et al*. ^404^ proposed a standardized PONV prophylaxis for ambulatory surgery based on patients’ Apfel risk-score (0–4): ondansetron (Apfel risk-score 2), additional dexamethasone (Apfel risk-score 3), and additional droperidol (Apfel risk-score 4). These investigators achieved low rates of PONV in ~90% of their patients, and PDNV was not a problem on the first day after surgery. In fact, pain after discharge was a much more common problem. Bergese *et al*. ^405^ found that triple therapy with scopolamine, ondansetron, and dexamethasone was an effective regime to prevent PONV in moderate- to high-risk patients undergoing craniotomy procedures. Dexamethasone and a 5-HT _3_ receptor antagonist combination has superior efficacy and thus it is recommended as the "ideal" choice for routine PONV prophylaxis ^406^. However, a study including same-day surgery patients with varying PONV risk factors revealed that adding ondansetron to a combination of low-dose droperidol and dexamethasone failed to increase antiemetic efficacy ^407, 408^. No difference was observed when comparing efficacy among combinations of 5-HT _3_ receptor antagonist with dexamethasone, 5-HT _3_ receptor antagonist with droperidol, or dexamethasone with droperidol ^409^. Interestingly, there was no reduction in PONV in the combinations containing metoclopramide compared to monotherapy alone ^410^.

As most patients undergoing surgery have one or two risk factors and 20–40% of these patients are expected to suffer from PONV if they do not receive a prophylactic antiemetic drug, combination antiemetic therapies have become increasingly vital in preventing PONV. Ideally, prophylaxis for PDNV would continue much further than the point of discharge from hospital or free-standing surgical care facility ^108, 109^. Recent research focused on different antiemetic drugs administered at various time points after surgery has evaluated the effects on reducing PDNV. For example, a study demonstrated that patients who received the combination of ondansetron (4 mg IV) and an oral disintegrating tablet of ondansetron (8 mg) immediately before discharge had less severe nausea and fewer vomiting episodes compared to IV ondansetron alone after discharge (3% versus 23%) ^411^. In a multi-center study, intraoperative dexamethasone did not appear to reduce PONV in the PACU but significantly reduced PDNV ^110^. Dewinter *et al*. ^412^ tested the effectiveness of a simplified algorithm for PONV prophylaxis with female patients receiving triple IV prophylaxis (dexamethasone and ondansetron plus either a target-controlled infusion with propofol or droperidol) and male patients received double prophylaxis with dexamethasone IV and ondansetron IV. This simplified algorithm for PONV prophylaxis resulted in a significant reduction in the incidence of PONV and better compliance with the PONV algorithm (46% versus 18%, *P* = 0.0001). Kumar *et al*. ^413^ compared a single dose of palonosetron 0.075 mg IV plus dexamethasone 4 mg IV to ondansetron 8 mg IV plus dexamethasone 4 mg IV (with ondansetron 4 mg administered every 8 hours IV for 48 hours) for PONV prophylaxis in post-chemotherapy ovarian cancer surgery patients receiving opioid-based patient-controlled analgesia (PCA). Ryu ^414^ found that palonosetron prophylaxis reduced the incidence and severity of PONV in high-risk patients undergoing total knee arthroplasty with a multimodal analgesia protocol consisting of spinal anesthesia, a continuous femoral nerve block, and fentanyl-based IV PCA. The increasing use of inexpensive, disposable acupressure devices (e.g. Relief Band, Pressure Right) should also be considered in patients at high risk for PDNV. In addition, these patients should be given instructions for appropriate “rescue” antiemetic treatment before they are discharged home. Recommendations for optimal antiemetic dosing when utilizing a combination “multimodal” therapy consisting of dexamethasone, droperidol, and ondansetron are as follows: ondansetron 4 mg IV, 4–8 mg of IV dexamethasone, and 0.625–1.25 mg of IV droperidol ^415^. Another study confirmed that low-dose granisetron (0.1 mg IV) in combination with dexamethasone (8 mg IV) is as effective as the combination of IV ondansetron (4 mg) and IV dexamethasone (8 mg) ^416^. Therefore, antiemetic drugs are now commonly administered at both the start and the end of surgery to patients considered to be at high risk of developing PONV ^417^. Adherence to validated PONV prophylaxis guidelines should be carefully evaluated before the patient is discharged from the PACU to guarantee that patients have received appropriate PONV prophylaxis during the perioperative period ^418^.

## Combined treatments for managing established PONV

Swift antiemetic management is mandated whenever PONV happens in patients who either did not obtain adequate prophylaxis or had ineffective prophylaxis. If PONV occurs in the immediate postoperative phase (within 6 hours postoperatively), despite antiemetic prophylaxis, an antiemetic belonging to a pharmacologic class other than that of the prophylactic drug regimen should be given. However, if the PONV occurs over 6 hours after surgery, it is suggested that a repeat dose of the original prophylactic is administered. If no prophylaxis was given, the recommended treatment is low-dose 5-HT _3_ antagonist (e.g. ondansetron 1–2 mg IV). Alternative treatments for active PONV include intravenous metoclopramide (10 mg), droperidol (0.625 mg), promethazine (6.25–12.5 mg), dolasetron (12.5 mg), granisetron (0.1 mg), palonosetron (0.075mg), or tropisetron (0.5 mg) ^419, 420^. Algorithms describing how to identify high-risk patients and how to guide the administration of multimodal treatments can significantly reduce the incidence of PONV and PDNV ^40, 421– 423^. Yazbeck-Karam *et al*. ^424^ investigated haloperidol versus ondansetron for the treatment of established PONV following general anesthesia and found that haloperidol (1 mg IV) was not inferior to ondansetron (4 mg IV) in the early treatment of established PONV. However, haloperidol was associated with an increased level of sedation ^423^. Hu *et al*. ^425^ combined a low dose of 2.5 μg/kg palonosetron with 15 μg/kg of droperidol and achieved a similar prophylactic effect as a higher 7.5 μg/kg dose of palonosetron compared to 7.5 μg/kg alone for treating emesis after eye surgery. For the treatment of existing PONV prior to discharge, a multimodal strategy should be considered because the recurrence rate of PONV over the subsequent 24 hours is 35–50% ^426^. A combination of low-dose ondansetron plus dexamethasone and droperidol or haloperidol has been found to be superior to monotherapy alone for the treatment of PONV in the PACU ^427^. Those interventions have proven to be effective for both prophylaxis for and treatment of PONV. Therneau *et al*. ^428^ found that the addition of aprepitant 40 mg PO to a multimodal antiemetic prophylactic regimen (triple antiemetic prophylaxis with dexamethasone, droperidol, and ondansetron) was associated with significant reduction of PONV during both the early recovery period and the first 48 hours postoperatively in patients undergoing bariatric surgery. Trimas and Trimas ^429^ concluded that a single dose of aprepitant 40 mg PO administered preoperatively can decrease the incidence of PONV in the early postoperative period after facial plastic surgery compared with ondansetron alone.

## Summary of current recommendations for reducing the risk of PONV and PDNV

The management strategy for each individual patient should be based on the level of risk for PONV, the patient’s pre-existing condition, patient preference, and cost efficiency. Patients should be informed about the potential consequences of PONV and PDNV during the preoperative evaluation. In addition to using a combination of antiemetic drugs with different mechanisms of action, the multifactorial etiology of PONV would also be best addressed by adopting a multimodal approach to pain management and minimizing baseline risk factors associated with PONV and PDNV in high-risk patients ( Table 3). Several effective strategies are recommended for reducing the baseline risk for PONV ( Table 6): (1) routine use of local anesthesia and regional anesthesia (e.g. local infiltration and/or peripheral nerve blocks); (2) propofol induction and maintenance infusion during general anesthesia and monitored anesthesia care (MAC); (3) minimization of perioperative opioid analgesics; (4) minimization of concentrations of volatile anesthetics; (5) minimization of the use of nitrous oxide and reversal drugs; and (6) ensuring adequate perioperative hydration ^17, 38, 52, 412, 430^. If general anesthesia is utilized, substituting a propofol infusion for maintenance of anesthesia in place of inhaled volatile anesthetics will reduce the risk of PONV. A combination of propofol and air/oxygen had additive effects, reducing the risk of early PONV by approximately 25% ^110^. The non-opioid analgesic drugs (e.g. nonsteroidal anti-inflammatory drugs [NSAIDs] like ketorolac or ketoprofen, cyclooxygenase-2 inhibitors [COX-2] like celecoxib or meloxicam, and acetaminophen [oral or intravenous]) should be part of a multimodal perioperative analgesic regimen ^33, 431– 435^. Pain and PONV after breast cancer surgery were more effectively reduced with a multimodal regimen utilizing non-opioid analgesics and antiemetics compared to a one- or two-component regimen ^433^. Similar results were found when using an opioid-free TIVA technique for bariatric surgery ^436^. Adequate preoperative and intraoperative IV fluid hydration is also an effective strategy for decreasing the baseline risk for PONV ^38, 269, 270^. Nitrous oxide had little impact when used for procedures lasting less than 1 hour ^437, 438^. Thus, nitrous oxide may be a good option for the short ambulatory procedures to facilitate a faster recovery from anesthesia. Although previous studies suggested that neostigmine produced dose-related increases in PONV ^51, 439^, a more recent study suggested that minimization of the neostigmine dosage failed to reduce the baseline risk of developing PONV ^440^. Sugammadex rapidly reverses the neuromuscular blockade caused by steroid-based muscle relaxants; it is a feasible alternative to neostigmine, edrophonium, or pyridostigmine in “at-risk” patients administered non-depolarizing muscle relaxants intraoperatively ^441^. Yağan *et al*. ^442^ reported that reversal with sugammadex 2 mg/kg (compared to neostigmine 50 μg/kg plus atropine 0.2 mg/kg) was associated with a lower incidence of PONV in the first hour after surgery and required less rescue antiemetic therapy in the first 24 hours after breast, strabismus, and middle ear surgery. Tas Tuna *et al*. ^443^ confirmed these results in patients undergoing elective laparoscopic cholecystectomy surgery. PONV prophylaxis is rarely warranted in low-risk patients. However, moderate-risk patients benefit from single or even often multiple antiemetic drug interventions. Use of two antiemetic interventions is recommended for adults and children at moderate risk, and three (“triple”) interventions should be administered to all high-risk patients ^38, 426^. The occurrences of PONV in patients who have received appropriate prophylactic antiemetic therapy should be treated aggressively using antiemetic drugs from a different pharmacologic class ^38, 444^. In patients who did not receive antiemetic prophylaxis, first consider using a generic serotonin antagonist. Do not repeat drugs used for prophylaxis until 6 hours have elapsed after completion of surgery. Do not repeat the use of transdermal scopolamine. If refractory symptoms persist, carefully evaluate for other causative factors such as excessive opioid use, draining blood into the gastrointestinal tract or nasopharynx, or gastrointestinal obstruction/ileus. Nonetheless, recognize that PONV/PDNV can still occur despite optimal prophylaxis in high-risk populations. Communication among the patient, anesthesiology team, surgical team, and perioperative nursing staff is essential for optimizing patient outcomes.

**Table 6.** Recommendations in relation to various risk factors of postoperative nausea and vomiting (PONV) following surgical procedures.

Mild risk (none or 1 risk factor)	Moderate risk (2 risk factors)	High risk (≥3 risk factors)
**No prophylaxis required or** **monotherapy with a cost-** **effective antiemetic drug** **if there is a risk of medical** **sequelae from PONV**	Choose a prophylactic combination of antiemetic medications When general anesthesia is needed, decrease pre- existing risk factors by reducing volatile anesthetic usage, use of opioids for analgesia, nitrous oxide, and elevated doses of reversal medications Use neuraxial anesthesia, peripheral nerve blocks, and infiltration of local anesthesia Utilize adjuvant nonpharmacologic options (e.g. acupressure and stimulation by electric acupoint)	Start therapy with two or three prophylactic medications that act on different receptors Minimize pre-existing risks by using opioid-reducing analgesia strategies Reduce the use of opioids in the perioperative period Reduce volatile anesthetic usage, use of opioids for analgesia, nitrous oxide, and elevated doses of reversal medications (e.g., naloxone, flumazenil, and neostigmine) Use neuraxial anesthesia, peripheral nerve blocks, and infiltration of local anesthesia
**Treatment options** **If prophylaxis fails or was not received, use antiemetic from different classes to prophylactic agent** **Re-administer only if >6 hours after post-anesthesia care unit; do not re-administer dexamethasone or scopolamine**		
